# Sources of Disease

**Published:** 1884-04

**Authors:** 


MEDICINE.

MONTHLY.

E. H. GIBBS, A.M., M.D........................EDITOR.

FOUNDED IN 1854.

### SOURCES OF DISEASE.

ALL Animal Life is a ceaseless alternation of death and repair, from birth till life closes. Exhalations and effete matters from the body are dead particles that may contaminate and destroy. The "breath of our nostrils" is rank poison; and, unmixed with the oxygen of the air, would be as destructive to animal life as the deadly "fire damp." Were these facts generally recognized and acted upon, there would not be one case of disease where there are now a hundred, and the average duration of human life would be advanced thirty years. To better illustrate our theory in its lowest extreme, let us look in upon one of the ten thousand miserable families occupying single rooms in tenement houses of this city. Here we find, huddled together, the parents and perhaps half a dozen children of various ages. They have not, usually, the intelligence to comprehend that "cleanliness is next to godliness," and if they had, they might not profit by the knowledge. In cold weather, they resist with an obstinacy worthy of a better cause, the admission of more out-door air than finds entrance through openings in the sash and cracks in the door, in spite of their efforts to keep it out. In this stifling room days and nights are passed. By springtime the whole family has become so poisoned with carbonic acid gas, that there is little power to resist disease; typhoid, perhaps, carries off the parents, and cholera infantum the younger children.

I am sorry to say that the difference between the condition of the family above described, and that of many people from whom we might expect better things, is only in degree —Who ever traveled in a railway car that was properly ventilated; or ever entered the crowded cabin of a ferry boat on a cold, damp day, without wishing the directors of the company might be compelled to make two or three trips a day under these circumstances! The time may come when ferry boats will be properly and sufficiently ventilated; until that time arrives it is safer to stand outside and incur the risk of taking cold, than to remain in the cabin and face the danger of typhoid fever or pneumonia. It seems to be quite generally admitted by physicians that consumption, as well as many other diseases, may be transmitted by the breath. Many cases of this kind have come under my own observation. I have now a patient afflicted with the disease, who undoubtedly contracted it from his wife whom he nursed in her illness. His health had always been good previously, and he remembers no case of consumption in his family. I do not think diseases are likely to be conveyed to others where ventilation is adequate. In the hospitals of Paris, the ventilation is excellent. I do not remember to have seen a small-pox patient separated from the others, during the years I was there. They occupied the same wards with other fever patients, and I do not think other persons contracted the disease in any of the wards.

In France, it is seldom that two persons occupy the same bed; whereas the custom is quite general in other countries. With good ventilation in the sleeping room, healthy children could sleep together without risk to health, perhaps; but it would be better for every person to have a separate bed. There has always been an idea that the weaker person profited by depleting vital power from his bed-fellow through some mesmeric influence or other obscure process. I am disposed to think there are good grounds for this belief. I think almost any one can recall instances where children who were under the care of aged and sickly nurses have suffered from the contact, and other cases where a sort of equilibrium or health compromise has established itself between a strong husbend and a delicate wife, or visa versa; so shat neither was in robust nor in failing health.

